# Wetland hydroperiod predicts community structure, but not the magnitude of cross-community congruence

**DOI:** 10.1038/s41598-020-80027-4

**Published:** 2021-01-11

**Authors:** Jody Daniel, Rebecca C. Rooney

**Affiliations:** B2-251, Department of Biology, University of Waterloo, Waterloo, ON N2L 3G1 Canada

**Keywords:** Community ecology, Wetlands ecology, Ecology, Ecology

## Abstract

A major focus in community ecology is understanding how biological interactions and environmental conditions shape horizontal communities. However, few studies have explored whether cross-community interactions are consistent or non-stationary across environmental gradients. Using the relative abundance of birds, aquatic macroinvertebrates and plants, we examined how cross-community congruence varied between short and long-hydroperiod prairie pothole wetlands in southern Alberta. These wetlands are structured by their hydroperiod: the length of time that ponded water is present in the wetland. We compared the strength of cross-community congruence and the strength of congruence between each horizontal community and wetland hydroperiod in wetlands that typically contain ponded water throughout the year to wetlands that dry up every summer. The strength of cross-community relationships was similar between more permanent and more ephemeral wetland classes, suggesting that biological interactions have a near equivalent role in shaping community composition, regardless of hydroperiod. However, because cross-community congruence, measured as the Procrustes pseudo-R value, was, on average, 77% ± SE 12% greater than that between each horizontal community and measures of wetland hydroperiod, we concluded that community structure is not shaped by hydroperiod alone. We attribute the observed cross-community congruence to (1) plants and aquatic macroinvertebrates influence birds through habitat and food provisioning, and (2) birds influence plants and aquatic macroinvertebrates by dispersing their propagules.

## Introduction

Understanding the mechanisms that explain the composition of biological communities is a major focus of community ecologists. Both environmental conditions and interactions between horizontal communities (i.e. sets of species sharing common needs in terms of resources or space)^1^ are known to dictate which species will establish in a given habitat^2–4^, and thus numerous studies have attempted to partition their relative influences on community composition^5–8^. However, apart from examination of the stress gradient hypothesis among plants^9–11^ and predation-permanence gradient model with aquatic macroinvertebrates and their predators^12^, only a few studies have explored whether the strength of biological interactions among multiple taxa is influenced by environmental conditions along a gradient^13–15^ beyond simply gradients in space or time^16,17^. Questions arising from this gap include: (1) do relationships among horizontal communities change along environmental gradients, and (2) does the strength of cross-community relationships vary with environmental conditions? By investigating whether the strength of cross-community relationships change across environmental gradients, we could better understand how communities assemble.

Because species differ in which environmental conditions are optimal for their growth and development, we may observe changes in the strength of interspecific cross-community interactions (herein referred to as non-stationarity) across environmental gradients^18–20^. Non-stationarity in cross-community relationships is widely reported in geographic space and across time^21,22^, and we would expect similar mechanisms as those deemed causal in studies of spatial or temporal gradients to explain non-stationarity in cross-community relationships along other environmental gradients. Indeed, such studies often attribute the spatial or temporal pattern to a correlated pattern in environmental conditions, though without explicitly quantifying those conditions. Across geographic space, we can attribute non-stationarity in plant-plant relationships to differences in environmental conditions under which species were able to establish^19^; the rate at which established species increased their abundances determined whether there was space for later-arriving species to also establish. The influence of differential tolerances and requirements among species on population structures has also been observed in predator–prey interactions. For instance, authors of one study argued that predation rates are lower in wetlands with shorter hydroperiods because fewer predators are able to sustain populations under these stressful conditions when the diversity and abundance of prey is lower^12^. Non-stationarity across environmental gradients could also be explained by species requiring additional defenses to combat new predators or competitors. For example, Alaska paper birch in nutrient-poor environments used carbon- vs. nitrogen-based defenses to herbivory, which resulted in them differing in their palatability to snowshoe hares across a gradient in soil chemistry^23^. More recent examinations of cross-community relationships across environmental gradients demonstrate non-stationarity between zooplankton and fish^24^, plants and insect herbivores^25^ and numerous other pairwise interactions^15^. Given these observations of non-stationarity of interspecific interaction outcomes along environmental gradients, we wanted to determine whether congruence between horizontal communities would be consistent across an environmental gradient. Or alternatively, whether one end of an environmental gradient might exhibit lower cross-community concordance than is evident at the other end of that gradient.

Congruence is a measure of the correlation between two multivariate matrices^26^. Typically, these comparisons are made between the relative abundance patterns evident in species belonging to different horizontal communities, commonly within a single taxon, to estimate the strength of inter-community interactions^27^ or between the pattern of relative abundances in one horizontal community and environmental conditions^28,29^ to estimate the strength of the dependency of a particular horizontal community on a given set of physicochemical factors.

**Figure 1:**
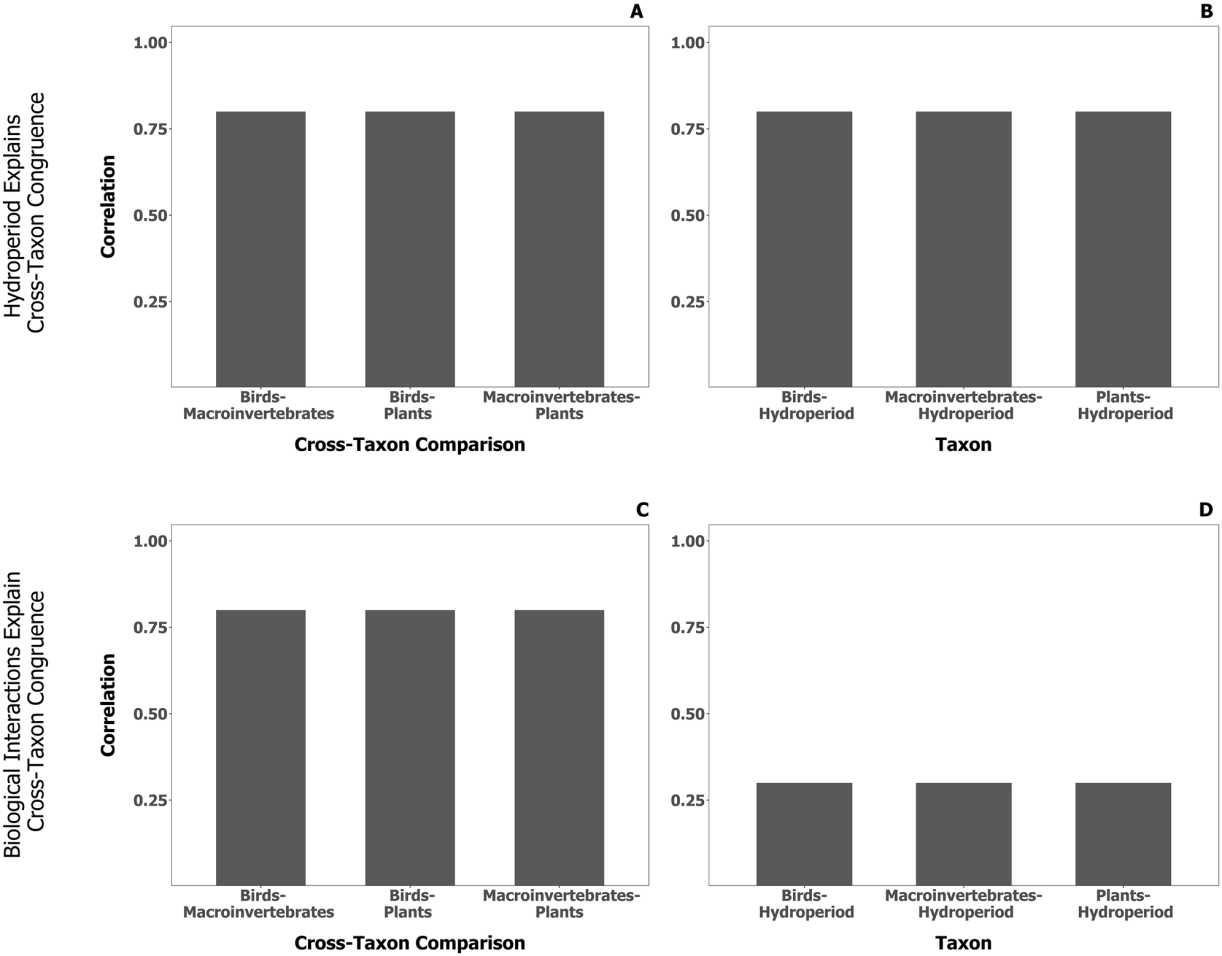
Hypothesized measures of congruence if cross-community congruence (**A**) was best explained by the influence of wetland hydroperiod (**B**), or if cross-community congruence was best explained by biological interactions (**C**) and not solely the influence of wetland hydroperiod (**D**). When the strength of cross-community relationships is similar in magnitude to that between each horizontal community and hydroperiod, we would expect that high cross-community congruence is explained by each horizontal community responding similarly to wetland hydroperiod (**A**, **B**). However, when cross-community relationships are much stronger than that between each horizontal community and hydroperiod, we would expect that high cross-community congruence is mostly explained by biological interactions (**C**, **D**).

While strong congruence between a horizontal community and some measure of environmental conditions in its habitat can indicate a structuring role of abiotic factors on community composition, strong cross-community congruence could be explained by either biological interactions^30^, horizontal communities responding similarly to a gradient in environmental conditions^31,32^, or a common biogeographic history among taxa^33,34^. If biological interactions are responsible for cross-community agreement among matrices of species’ relative abundance, we expect that cross-community congruence will exceed the strength of congruence between either horizontal community and a matrix of environmental variables, particularly if the system is characterized by a simple dominant environmental gradient (Fig. 1A,B). In contrast, if the strength of cross-community congruence is equal to or less than the strength of congruence between a matrix of environmental variables and one of species’ relative abundances for a given horizontal community, then we must concluded that the cross-community congruence we observe could be attributed to a common response to environmental conditions or even a common biogeographic history^35^ (Fig. 1C,D), biogeography and environmental conditions being typically correlated and difficult to partition.

This approach to evaluating the relative importance of cross-community interactions and environmental filtering on community composition is best implemented in a system that is largely structured by a single environmental gradient (e.g., a moisture-aridity gradient such as that created by variation in inundation time in wetlands or precipitation in desert ecosystems) because differences in the species pools between habitats can be explained by their response to the environmental gradient^36^. Along such a gradient, the availability of water can act as a clear environmental filter, excluding taxa that lack adaptations to persist under either dry or inundated conditions^37^. This results in the emergence of distinct communities, dependent on moisture availability^38,39^. Prairie pothole wetlands, for example, differ in the diversity and community composition of birds, aquatic macroinvertebrates and plants along a gradient in hydroperiod from ephemeral to permanently-ponded^36^. Hydroperiod in these wetlands influences whether a wetland supports only wet meadow species or includes more water-loving robust emergent species like cattails and bulrushes or even submersed aquatic and floating vegetation^40^. Hydroperiod also dictates if a wetland will support aquatic macroinvertebrates that cannot survive dry-down events or if such taxa will be excluded^37^. Since the foraging and nesting opportunities of migratory birds are determined by wetland vegetation characteristics and the availability of aquatic macroinvertebrate prey, hydroperiod also indirectly dictates bird community composition^41^. Given these three taxa exhibit distinct communities at different positions along the hydroperiod gradient, we can test whether the strength of cross-community congruence differs between short and long-hydroperiod prairie potholes.

In an earlier study, beta diversity was inversely correlated with wetland hydroperiod for wetland birds, aquatic macroinvertebrates and plants^36^. The authors speculated that species in wetlands with longer hydroperiods had more time to progress toward community equilibrium through interspecific interactions, whereas community composition in wetlands with brief hydroperiods was more a product of ecological drift. If this hypothesis were correct, we expect to see weaker cross-community congruence in short-hydroperiod than long-hydroperiod prairie pothole wetlands. We consequently asked three questions, using relative abundances of three horizontal communities (birds, aquatic macroinvertebrates and plants): examining species from prairie potholes ranging from short- to long-hydroperiods (1) is there significant congruence among these three horizontal communities; and (2) if so, does a common response to the dominant environmental gradient (hydroperiod) alone explain the observed cross-community congruence; then, after partitioning the dataset into long- and short-hydroperiod prairie potholes, (3) is there evidence of non-stationarity in cross-community congruence between prairie potholes of long vs. short-hydroperiod wetlands?

## Materials and methods

### Study area

**Figure 2:**
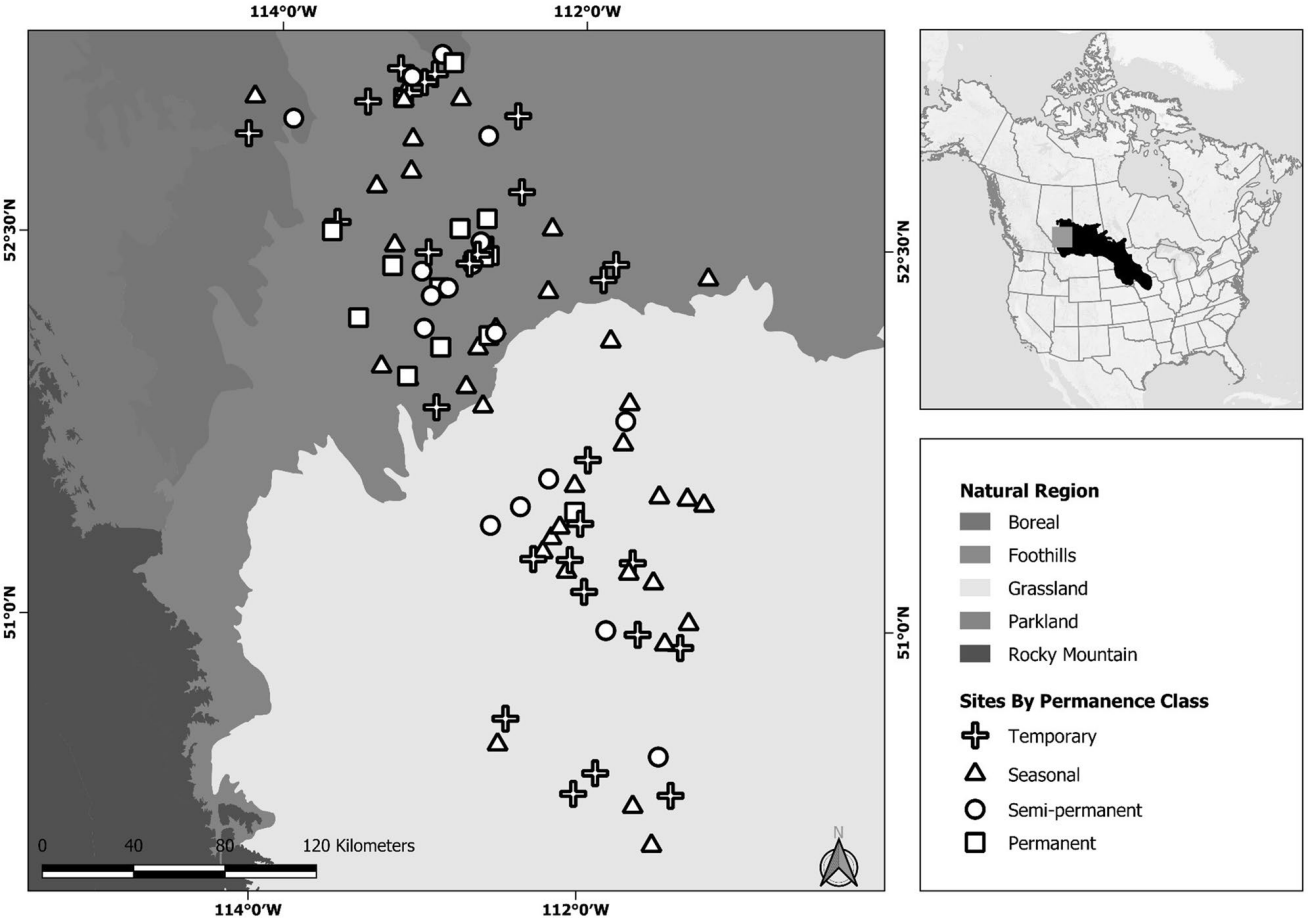
Map of study our region, situated in the northern prairie pothole region (inset map). Our 96 wetland sites covered the Grassland and Parkland Natural Regions, and represented temporary (*n* = 28), seasonal (*n* = 35), semi-permanent (*n* = 17), and permanent (*n* = 14) ponded-water permanence classes.

Our study took place in the Grassland and Parkland Natural Regions of Alberta, Canada (Fig. 2). Wetlands in the region are called prairie potholes and comprise water-filled depressions that were formed in the last glacial period^42^. The climatic conditions are semi-arid since annual precipitation exceeds evapotranspiration rates^43^. While mixed-grass prairie dominates the Grassland Natural Region, both deciduous forest and prairie are widespread in the Parkland Natural Region^44^.

### Study design

Our 96 study sites spanned a gradient in hydroperiod, i.e., they ranged in pond permanence from temporary to permanent^40^*.* Further, they were selected to represent the size-frequency distribution of wetlands within their respective sub-watersheds, based on the Alberta Merged Wetland Inventory^45^. As such, most were small (mean size 0.81 ± 0.12 ha). Importantly, wetlands classified as short- vs. long-hydroperiod were equivalent in their size (t-test: t = 0.187, df = 90.4, p-value = 0.852).

### Biological surveys

#### Birds

Birds were surveyed using both visual and auditory surveys, twice during the peak breeding season (May–June in either 2014 or 2015). Importantly, species abundances were summed across visits, rather than averaged, to account for the staggered breeding seasons among species. More details on these bird surveys are provided in another study^46^. In brief, surveys commenced half an hour before sunrise and went no later than six hours thereafter. Surveys were rescheduled if weather conditions were unfavorable to bird activity (e.g., rain, traffic sounds, or wind enough to rustle field notes). First, 10-min long visual surveys from a vantage point that covered the entire open water zone were undertaken to record any foraging or nesting birds before the site was entered, as observers entering the site can flush waterfowl. Next, observers conducted auditory surveys that were 8-min long, 100 m fixed-radius point counts, typically carried out at the center of the wetland. In larger wetlands (> 3 ha), multiple auditory surveys were carried out, spaced > 100 m from any wetland edge and > 200 m from any other point count location to ensure independence. Counts across multiple auditory point counts within a wetland were summed to reflect differences in wetland size. The identity and abundance of species detected by visual and auditory survey techniques was recorded (species list in Supplemental Material 1). We ensured not to double count individuals that were recorded during the visual survey in the point count surveys, or birds that relocated between point count surveys, where multiple point counts were warranted by the size of the wetland.

#### Aquatic Macroinvertebrates

Aquatic macroinvertebrates were sampled during the same period as birds, using the quadrat-column-core method^47^, which was revised^48^ for use in our study region. Sampling was stratified between the open-water zone (submersed and floating vegetation) and the emergent zone (cattail, bulrush, or other robust perennial sedges), presuming both zones were present. Three replicates of each sample type were collected in each wetland zone: (1) a 10 cm deep, 4.8 cm diameter sediment core, collected using a steel corer; (2) a 0.25 m^2^ vegetation sample, clipped from the emergent or submersed vegetation and then washed vigorously to remove clinging invertebrates; and (3) two, 10 cm diameter water column samples obtained using a tube-sampler inserted to just above the sediment. The replicates of each sample type were composited, yielding a single water column, sediment core, and vegetation sample per wetland vegetation zone (open water and emergent). These were then sorted to remove aquatic macroinvertebrates so they could be identified to the lowest practical taxonomic level (typically Family)^49,50^. For vegetation samples, we used a Marchant box to sub-sample based on the protocol of the Canadian Aquatic Biomonitoring Network^51^, where the taxon abundances were area-weighted to estimate density per meter-squared. Similarly, counts from water samples were scaled to the meter squared, and then water and sediment densities were summed to represent each wetland zone, and averaged across zones to obtain wetland-level data on invertebrate relative abundances. Ultimately, sediment core sample fractions were excluded from analysis because: (1) densities were low and (2) there were no taxa in the sediment cores that were novel to the combined water column and vegetation samples. A comprehensive list of taxa observed is provided in Supplemental Material 1.

#### Plants

Plant surveys occurred in late July to August, which coincided with peak aboveground biomass and when most herbaceous species could be confidently identified. First, the extent of each plant assemblage was mapped, and this was based on their vegetation structure (e.g., deciduous tree, coniferous tree, dead deciduous, dead coniferous, deciduous shrub, coniferous shrub, robust emergent, narrow-leaved emergent, forb, broad-leaved emergent, floating-leaved vegetation) and then by co-dominant or dominant species. These extents were determined in the field by mapping the assemblage boundaries with a GPS/GNSS unit with sub-meter real-time accuracy (SX Blue II receiver, by Geneq Inc., Montreal, Canada). For each 100–5000 m^2^ sized community, the identity and percentage cover (modified Braun-Blanquette approach) of each vascular plant species within five, 1 m^2^ quadrats were recorded. For communities larger than 5000 m^2^, an additional quadrat was surveyed per 1000 m^2^ of community area over the 5000 m^2^ threshold. In addition to vascular plants, the identity and percentage cover of the following classes were also included: algae, bare ground, litter, moss, rock, seedling/unidentified forb, standing dead litter, and open water (species list in Supplemental Material 1). Note, only the percent cover of vascular plants was included in subsequent analyses. More details on these plant surveys are provided in another study^52^.

#### Hydroperiod

We consider hydroperiod a latent variable that is indicated by several measurable variables such as the approximate number of days the wetland contained ponded water, the maximum water depth, the ratio of water amplitude to the maximum water depth, or an index of evaporative loss based on stable isotope analysis. In our study, we used these four measurements to approximate hydroperiod. At each wetland, we installed a staff gauge in May at the deepest point of the open water zone. We collected water depth measurements from these staff gauges every 3–5 weeks between May and September. If the wetland dried out entirely, we recorded the date that this was first observed. If the wetland remained flooded until September, then we considered it to possess ponded water for 365 days. The difference between our deepest and shallowest water depth measurement was the wetland’s amplitude. Lastly, we collected 30 mL water samples from each wetland in May, which was later used to estimate evaporative loss. For details on the stable isotopes analysis, see^53^.

### Statistical analysis

#### Congruence

To determine if we could (1) detect cross-community congruence, (2) attribute cross-community congruence to a dominant environmental gradient (hydroperiod) and (3) detect a difference in the strength of congruence in short vs long-hydroperiod wetlands, we used a Procrustes analysis. In terms of our general approach, we first measured the strength of congruence between each pair of horizontal communities: (1) birds and plants, (2) birds and aquatic macroinvertebrates, and (3) plants and aquatic macroinvertebrates. Next, we measured congruence between each horizontal community individually and the four measures of wetland hydroperiod. Then, we compared cross-community congruence and congruence between each horizontal community and measures of wetland hydroperiod to determine whether a common response to variation in hydroperiod could explain any observed cross-community congruence (Fig. 1C,D). Next, to test for non-stationarity in any observed cross-community congruence in short vs. long-hydroperiod wetlands, we sub-divided the dataset into wetlands of low (temporary and seasonal) permanence class and wetlands of high (semi-permanent and permanent) permanence class and then recalculated both cross-community congruence and congruence between each horizontal community and the matrix of hydroperiod indicators. We then compared the strength of these congruence measures between the low and high permanence class wetland subgroups.

Though the Mantel test is popularly used to measure congruence in ecology^26^, Peres-Neto and Jackson showed that Procrustes analysis is better at detecting significant relationships (lower risk of type II errors). Additionally, in a majority of studies testing for multivariate correlations, Procrustes analysis can be substituted for a Mantel test^26^, apart from when the aim is to test for a relationship between community dissimilarity and geographic distance^55^. To measure congruence in a Procrustes analysis, matrices are rotated and translated until finding the lowest possible variance that still maximizes their fit in Euclidean space^54^. As a measure of fit, we use the sum of square residuals between the matrices in their optimal configurations and a pseudo-R value^54^. Another advantage of the Procrustes analyses is that it is insensitive to differences in dimensionality between matrices. Zero-filled columns are added to the smaller matrix to match dimensions of the larger matrix, and this is unlikely to affect measures of congruence^26,56^. Given our aim to assess how congruence changes with pond permanence, we were confident that Procrustes analysis was most appropriate for our study, which was implemented using the protest function in the vegan package^57^. For each horizontal community, we applied a Hellinger transformation to our relative abundance matrices to make our data suited for projection in Euclidean space^26^, as required by the Procrustes analysis.

### Rarefication

Sample size can influence estimates of congruence^58^. To ensure our sites were representative of the frequency distribution of permanence classes in our wetland inventory, we sampled an unequal number of wetlands across permanence classes (Fig. 2). Unequal treatments and small sample sizes can cause: (1) higher than expected estimates of congruence for classes with spatially aggregated wetlands (e.g. permanently-ponded wetlands), or (2) an inability to detect congruence in classes with fewer wetlands. To determine if sample size influenced our ability to detect congruence, or the magnitude of congruence, we rarefied our data to identify the sample size threshold at which the sensitivity to sample size plateaued. First, we subsampled our Hellinger-transformed relative abundance matrices, increasing *n* from 3 to 40 and selecting the same sites from each horizontal community in the cross-community comparison (birds versus macroinvertebrates, birds versus plants, macroinvertebrates versus plants). Then, for each subsample and cross-community comparison, we measured congruence. Since congruence can be influenced by this random subsampling, we repeated the rarefication 100 times. We repeated these sensitivity tests in analyzing congruence between each horizontal community and our matrix describing hydroperiod. We found the mean and standard error across iterations for the 3 to 40 subsampled sites (Supplemental Material 2). On average, changes in congruence were marginal with > 23 sites. Thus, we combined the temporary and seasonally classified wetlands (short hydroperiod: *n* = 65) and our semi-permeant and permanently classified wetlands (long hydroperiod: *n* = 31) into separate groups, such that this threshold was exceeded.

The long-hydroperiod group still constituted fewer sites (*n* = 31) than the short-hydroperiod group (*n* = 65), which could bias our comparison of congruence between wetland permanence class groups. To counter this bias, we stratified our horizontal community relative abundance matrices and the matrix describing hydroperiod by permanence class. Using the package fifer^59^ in R, we then subsampled and randomly selected 31 sites (without replacement) from the (1) entire dataset and (2) short-hydroperiod wetlands (including both seasonally-ponded and temporarily-ponded wetlands). We compared this to all 31 long-hydroperiod wetlands (including both semi-permanently ponded and permanently-ponded wetlands). We repeated this random sampling 1000 times, measuring congruence for each iteration.

## Results

### Congruence

**Figure 3:**
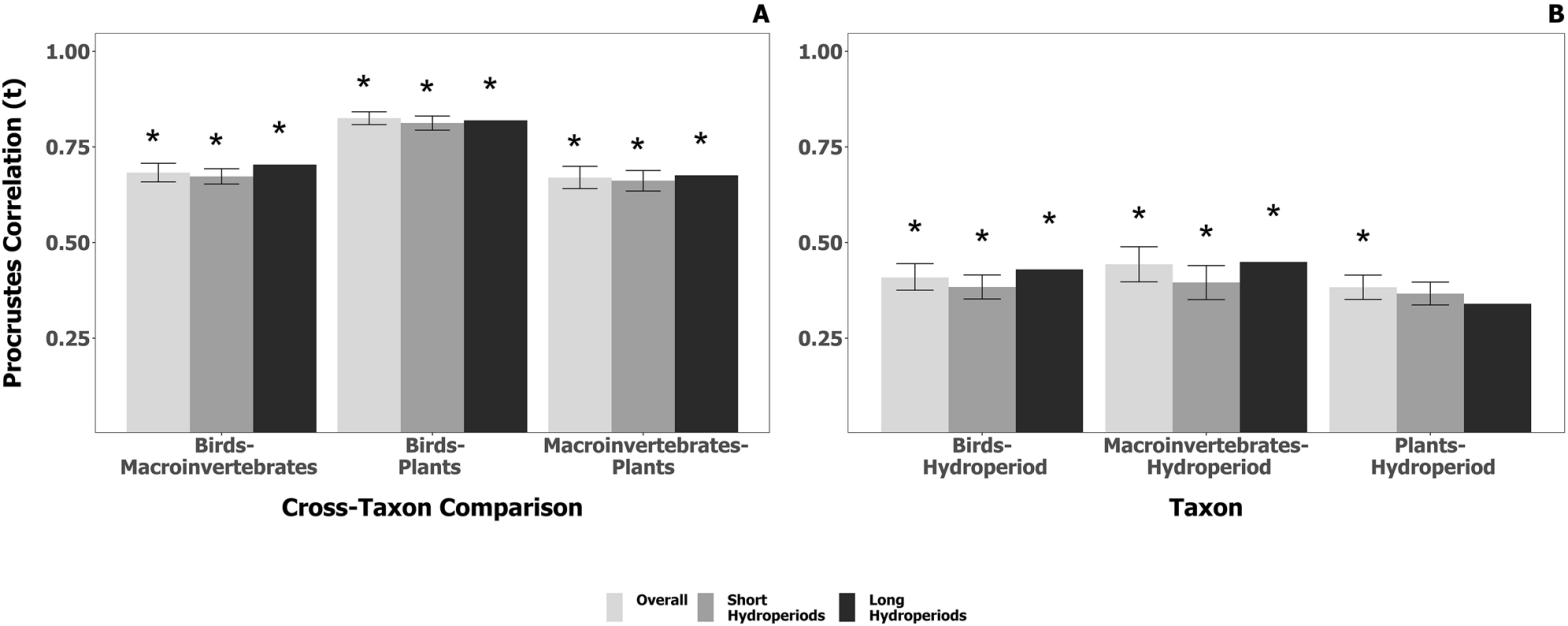
Congruence among horizontal communities (**A**) and between each horizontal community and hydroperiod (**B**) using a Procrustes Analysis. We measured congruence for our balanced rarefied datasets at 31 sites. Over 1000 iterations, we sampled without replacement: (1) all sites, regardless of permanence class (*n* = 96) and (2) short-hydroperiod wetlands (including both seasonally-ponded and temporarily-ponded wetlands; *n* = 65); thus, error bars are 90% confidence intervals from bootstrapping. Long-hydroperiod wetlands were inclusive of those that were semi-permanently and permanently ponded; “*” indicates that congruence was significantly greater than zero (*p*-value < 0.1).

The strength of cross-community congruence (Fig. 3A) was much larger than that between each horizontal community and measures of hydroperiod (Fig. 3B), when we consider all wetlands surveyed. Using the Procrustes pseudo-R value, the strength of bird cross-community relationships (i.e., bird-aquatic macroinvertebrate, bird-plant) were 84% higher in magnitude than that between birds and hydroperiod. With aquatic macroinvertebrates, relationships with both birds and plants were 52% larger in magnitude than aquatic macroinvertebrate-hydroperiod relationships. Similarly, for plants, cross-community relationships were 95% larger in magnitude than that between plants and hydroperiod. For a full list of Procrustes pseudo-R values and the associated *p*-value, see Supplemental Material 3.

Differences in congruence based on permanence class (short hydroperiod: temporary and seasonal; long hydroperiod: semi-permanent and permanent) were marginal, contrary to our prediction. While birds and macroinvertebrates had marginally stronger congruence with hydroperiod in long-hydroperiod wetlands (Fig. 3B), difference in cross-community congruence between the long and short hydroperiod wetlands were negligible.

## Discussion

Biological interactions are important drivers of community composition and some argue that biological interactions are equally as important in shaping community composition as are environmental filters^2,3^. Without understanding the relationship between one horizontal community and another, we have an incomplete understanding of how communities assemble. It is difficult to disentangle the relative influence of biological interactions and environmental filters on community composition in the absence of manipulative experiments^60,61^, especially with the use of natural experiments as opposed to causal-based models (e.g., structural equation models)^5,62^. However, our results confirm that hydroperiod alone cannot explain patterns in species relative abundances, despite sampling across a strong hydroperiod gradient, since there were stronger relationships among the horizontal communities we surveyed than between each horizontal community and our measures of hydroperiod. Similar to our findings, a Norway study reported a strong relationship between biological interactions and plant biomass in alpine grasslands along a precipitation gradient^63^ and a highly relevant Brazilian study of floodplain wetlands also found a relationship between hydroperiod and woody plants^64^. Interestingly, although the authors observed a high congruence among the horizontal communities that they studied, including birds, plants and spiders, they concluded that horizontal communities evidencing the highest concordance were more strongly associated with the same environmental factors, such as hydroperiod and flood intensity.

Interestingly, though cross-community congruence was high compared with the congruence between each horizontal community and the matrix of hydroperiod indicators, we did not detect a difference in the strength of cross-community congruence between wetlands of short and long hydroperiods. We attribute our failure to detect this non-stationarity in the strength of cross-community relationships to two factors. First, out of the three horizontal communities that we studied, hydroperiod had the strongest relationship to aquatic macroinvertebrates, filtering out those invertebrates missing the capacity to survive drawdown and desiccation. Therefore, it is likely that the influence of vegetation on the abundance of macroinvertebrates is masked by the stronger influence of hydroperiod^37^. Work in Australian ephemeral wetlands suggests that the influence of hydroperiod on aquatic macroinvertebrates may be similar to that on plants, and authors reported that the duration of inundation and water depth will dictate which plants will establish^65^. Consequently, from short to long-hydroperiod wetlands, we should observe plant and aquatic macroinvertebrates that are adapted to the same conditions, and this would mean that plants will have the same magnitude of influence on aquatic macroinvertebrates across the hydroperiod gradient. Second, birds select wetlands based on whether their foraging and nesting needs can be met^66^ (traits listed in Supplemental Material 4), which is typically dependent on vegetation in both short and long-hydroperiod wetlands. As an example, our long-hydroperiod wetlands were occupied by waterbirds, whereas our short-hydroperiod wetlands were more associated with upland birds (Supplemental Material 5A.D). Thus, while we may observe birds that feed on aquatic plants/insects or nest in reeds in long-hydroperiod wetlands, birds in short-hydroperiod wetlands likely had foraging and nesting behaviors suitable for the prey and nesting habitat available. In other words, the birds are cueing to vegetation in their selection of wetland habitat across the measured hydroperiod gradient.

For wetland biota that are active dispersers, cross-community biological interactions may be more important in shaping their community composition than for sessile species. We demonstrate that bird abundances were most strongly tied to plant and aquatic macroinvertebrate abundances, and birds were the strongest dispersers of the horizontal communities we studied. Birds in the NPPR are migratory, and they are known to choose wetlands for pairing and brood rearing based on the vegetation structure within the landscape and wetland-scale vegetation characteristics^67^. Thus, birds occupying the wetlands we surveyed actively selected these areas because their foraging and nesting needs could be met. For aquatic macroinvertebrates, however, some families are able to colonize neighboring wetlands with better-suited hydroperiod regimes, when in their adult phases; for those invertebrates incapable of moving between wetlands (i.e., those in their aquatic stage), drawdown may extirpate them from a wetland, if they do not have desiccation-adapted traits^68,69^. Consequently, because aquatic macroinvertebrates are not generally able to select wetlands based on their preferred hydroperiods, plants may have a smaller role in structuring their communities than for birds. For plants, which are passive dispersers, the water depth gradient may determine which subset of the seedbank will germinate at a given location^65,70^ (traits in Supplemental Material 4). Because plants in these short-hydroperiod wetlands had seeds that are typically animal dispersed (Supplemental Material 5C,F), we can conclude their abundances may also be influenced by birds. Seed dispersal by birds is widely reported to influence wetland plant abundances^71–74^; cyclic drying increases seedbank diversity as the sediment is frequently exposed^75,76^. However, the authors also argue that seedbank composition and richness are congruent along the water depth gradient; when comparing seedbanks between wetlands, they are often indistinguishable. This would suggest that while seed dispersal by birds can influence seedbank diversity, it is hydroperiod that determines which plants within the seedbank establish^65^. Though plant abundances are related to seed dispersal by birds, the strong filtering of hydroperiod on their abundances results in birds being more sensitive to cross-community interactions as they can select which wetland meets their foraging and nesting needs.

There are caveats that warrant consideration in explaining the weaker congruence between each horizontal community and hydroperiod than among the horizontal communities. First, our vegetation surveys focused on emergent and meadow species, not submersed aquatic vegetation in the open water, which in short-hydroperiod wetlands is often gone by August. Submersed aquatic vegetation is characteristic of wetlands with longer hydroperiods^40^. Thus, incorporating submersed aquatic vegetation could increase the strength of the plant-hydroperiod congruence. However, because congruence between hydroperiod and the other horizontal communities (i.e., birds and aquatic macroinvertebrates) were similar in magnitude to that of plants, we believe that exclusion of submerged plants did not overly affect our results. Another factor that could have diminished our measures of congruence between each horizontal community and hydroperiod is that the variables we used to measure hydroperiod, such as permanence class, are proxies of the duration of inundation and the persistence of ponded water in the wetlands. However, there was strong agreement among the proxies across a gradient in Natural Region, climate and land use, evidencing that they are robust indicators of hydroperiod in our study^77,78^.

## Conclusion

Contrary to our predictions, we did not detect non-stationarity in cross-community relationships across an environmental gradient. This was surprising because we hypothesised that longer hydroperiods would facilitate more time for cross-community interactions, and our failure to detect any differences suggests that horizontal communities in short to long-hydroperiod wetlands are equally related to each others’ abundances. Secondly, we detected stronger correlations between horizontal communities than between each horizontal community and measures of hydroperiod, and this was strongest for birds and plants. We hypothesize that these stronger correlations suggest that plant abundances are important in determining whether a bird will occupy a wetland, and that birds likely influence plant abundances when they disperse their seeds.

## Supplementary Information

Supplementary Information 1.

Supplementary ESM 1.

Supplementary ESM 3.

Supplementary ESM 5.
