# Supplementary material for: Wetland hydroperiod predicts community structure, but not the magnitude of cross-community congruence
Source: Sci Rep. 2021 Jan 11;11:429. doi: 10.1038/s41598-020-80027-4 (PMC7801406; doi:10.1038/s41598-020-80027-4)
Supplement: Supplementary file 1 — Supplementary Information 1. [file 41598_2020_80027_MOESM1_ESM.docx]

**Title:** Wetland hydroperiod predicts community structure, but not the magnitude of cross-community congruence.

**Authors**: Jody Daniel^1^ and Rebecca C Rooney^1*^

^1^B2-251, Department of Biology, University of Waterloo, Waterloo, Ontario, Canada, N2L 3G1

***Corresponding Author**: **Phone** – 519-888-4567 EXT 33820; **Email** – [rebecca.rooney@uwaterloo.ca](mailto:rebecca.rooney@uwaterloo.ca)

**Author Affiliations and Email Addresses**^[[1]](#footnote-1)^:

Jody Daniel: University of Waterloo, Department of Biology. [j9daniel@uwaterloo.ca](mailto:j9daniel@uwaterloo.ca)

ORCID: 0000-0003-3153-8164

Rebecca C. Rooney: University of Waterloo, Department of Biology. rrooney@uwaterloo.ca ORCID: 0000-0002-3956-7210

**Supplemental Material** **1** – Code, scientific name, and common name of bird (sheet 1), aquatic macroinvertebrate (sheet 2) and plant (sheet 3) taxa observed at our 96 wetland sites.

**

Supplemental Material** **2** – Measures of congruence for *n* = 3 to 40 randomly selected sites, averaged across 100 iterations. Regardless of *n,* we measured congruence between the same sites, either between horizontal community (A – C) or between each horizontal community and our proxies for hydroperiod (D – F). Here, we show the mean and standard error. Because of computational time, we limited the number of iterations to 100, though we used 1000 iterations in all other analyses requiring permutation. With a larger number of iterations, we expect smaller errors, but the same trends in the mean.

**Supplemental Material** **3** – Results from our Procrustes analysis on our balanced, rarefied dataset for the 1000 iterations, which include: 1) cross-community comparisons (sheet 1) and 2) comparisons between each horizontal community and our proxies for hydroperiod (sheet 2).

**Supplemental Material** **4** – Trait values and descriptions used in estimating total abundance of species/families by functional traits for the nonmetric multidimensional scaling with birds (worksheet 1 – 2), macroinvertebrates (worksheet 4 – 5) and plants (worksheet 6 – 7).

**
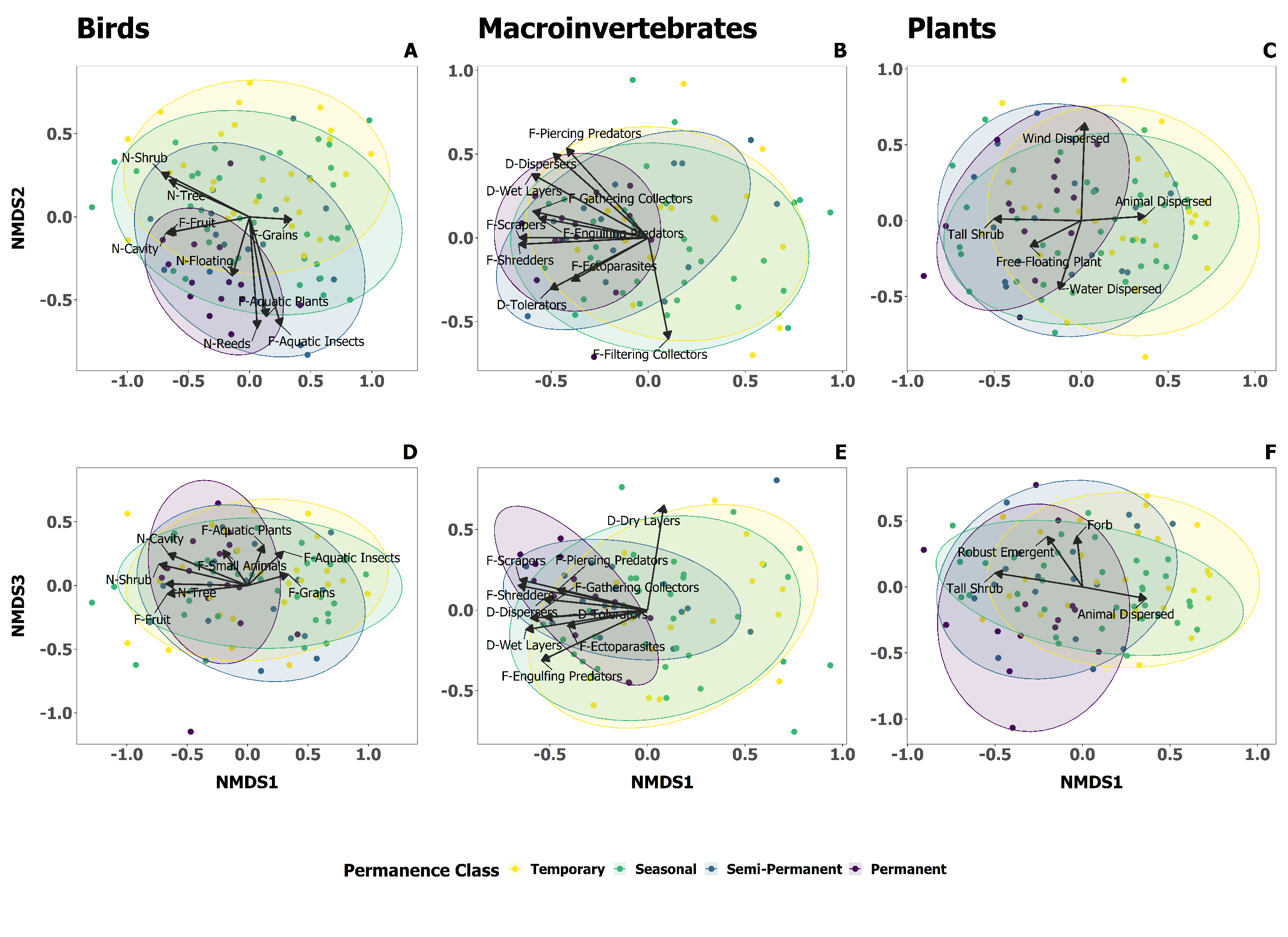
**

**Supplemental Material** **5 –** Three-dimensional nonmetric multidimensional scaling (NMDS) analysis of birds, macroinvertebrates and plants at our 96 study wetlands, with sites grouped by permanence class. Before implementing the NMDS, we square-root transformed our bird and macroinvertebrate data. For plants, we used an arcsine square-root transformation. Following, we relativized the transformed community relative abundances by their respective column maximums. Next, to implement the NMDS, we used the “metaMDS” function under the vegan package (Oksanen et al. 2017)^[[2]](#footnote-2)^ in R. We used Bray-Curtis dissimilarity and allowed the algorithm a maximum of 100 tries to find two convergent solution. Before implementing the final NMDS, we ran the function 100 times, increasing the number of axes from one to ten. Thereafter, we used a screeplot to determine the optimal number of axes, which was specified in the final NMDS. For all communities, stress was moderate (birds – 17.03, macroinvertebrates – 17.16, plants – 19.16), and the NMDSs stabilized in fewer than 50 iterations (birds – 47, macroinvertebrates – 20, plants – 41). We included vectors on the total abundance of species/families by functional traits (see Supplemental Material 5, for a full list of functional traits) with correlations that were greater than 0.1 for one or both axes. Vectors shown indicate feeding behaviours/primary diet (F), nesting habitat (N), desiccation strategy (D), plant wetland indicator status and plant seed dispersal mechanism. The ellipses are 90 % confidence intervals for sites by permanence class. The hydroperiod gradient is shown on NMDS 1 for aquatic macroinvertebrates and plants and NMDS 2 for birds.

1. **Declaration of Authorship**: RCR designed the study; RCR collected the data; JD analyzed the data; JD drafted the manuscript; JD and RCR revised the manuscript and discussed ideas. [↑](#footnote-ref-1)
2. Oksanen J, Blanchet FG, Kindt R, et al (2017) vegan: Community Ecology Package. R Packag. version 2.4-2 1 [↑](#footnote-ref-2)
